# A rare case report of neuro-brucellosis with concurrence of depression, visual impairment, bilateral sensorineural hearing loss, and paraplegia

**DOI:** 10.1371/journal.pntd.0012824

**Published:** 2025-07-29

**Authors:** Hossein Pourmontaseri, Maziyar Rismani, Barbad Karami, Zahra Montaseri, Ali Azmi, Sedighe Hooshmandi

**Affiliations:** 1 Student Research Committee, Fasa University of Medical Sciences, Fasa, Iran; 2 Trauma Research Center, Shahid Rajaee (Emtiaz) Trauma Hospital, Department of Neurosurgery, Shiraz University of Medical Sciences, Shiraz, Iran; 3 Department of Infectious Diseases, School of Medicine, Fasa University of Medical Sciences, Fasa, Iran; 4 Medical Imaging Research Center, Shiraz University of Medical Sciences, Shiraz, Iran; George Washington University School of Medicine and Health Sciences, UNITED STATES OF AMERICA

## Abstract

Neurobrucellosis is a rare manifestation of brucella infection, which would be life-threatening and result in multiple disabilities. Brucellosis commonly manifests with fever, arthralgia, and myalgia. Moreover, most patients with Neurobrucellosis present with significant lesions in the brain, spinal cord, or vertebral column; some cases show no lesions in their magnetic resonance imaging (MRI). The present case is a 32-year-old rural man with suspicious contact with animals at his work who was presented with ataxia, paraplegia, and urine-fecal incontinence without typical symptoms of brucellosis. Broad-spectrum diagnostic methods for neurobrucellosis were employed, including the Wright and 2-mercaptoethanol (2-ME) tests on plasma and brain, as well as spinal MRI. However, no significant pathologies were found in the brain or spinal imaging that could explain the patient’s clinical condition, and the Wright and 2-ME tests were negative. Also, despite a comprehensive approach to different viral, bacterial, autoimmune, systemic, metabolic, and organic etiologies, the symptoms of the patient got worse, and he experienced depression, sensorineural hearing loss (SNHL), and visual impairment in the following months. Eventually, the patient’s cerebrospinal fluid specimen Wright & 2-ME tests became positive, and a standard antibiotic regimen, including doxycycline, rifampin, and ceftriaxone, was administered for several months. In his last follow-up two years later, all neurological and psychological issues had disappeared except mild ataxia and hearing impairment. Hence, the prozone phenomenon should be considered in the false-negative Wright tests in endemic regions for brucellosis.

## Author summary

Maziar Rismani, Hossein Pourmontaseri, Barbad Karami, and Ali Azmi are medical students at Fasa University of Medical Sciences, working on a range of subjects, including neurological and Endemic Tropical Infectious Diseases. Our Corresponding Author, Dr. Zahra Montaseri, detected the following case due to her keen clinical understanding, providing the patient with the best possible clinical care. Dr. Montaseri also offered the chance to follow up on the case after the medical therapy was completed.

## Introduction

Brucella, the causative agent of brucellosis, is a well-known zoonotic bacterium that contaminates several species of pigs, sheep, goats, cattle, and dogs [1]. The main routes of contamination include direct contact with animals or their feces or urine, and consumption of dairy products [2]. Pasteurizing contaminated products before consumption is a safe way to decrease contamination, although screening and timely treatment of animals would be the best way to prevent brucellosis [3].

Brucellosis damages various regions, such as the lungs, joints, spleen, lymph nodes, and reproductive organs [4]. Neurobrucellosis is a relatively rare infection that presents with headache, fever, sweating, nausea, and vomiting, and is similar to many other central nervous system (CNS) infections [5]. Neurobrucellosis is accompanied by complications such as arthralgia, tremors, hearing loss, visual disturbance, and low back pain [5]. Imaging of the CNS shows the involvement of white matter, vascular parts, cerebral edema, and inflammation of the affected regions in patients with Neurobrucellosis [6]. Wright and 2ME cerebrospinal fluid (CSF) tests are commonly used to detect microorganisms in Neurobrucellosis cases [7].

The first and most crucial step in detecting Neurobrucellosis on time is taking a detailed history and an accurate physical examination. Due to the prozone phenomenon, the Wright test in higher dilutions should also be considered. The present study reports a case of neurobrucellosis infection with uncommon presentations, characterized by a negative initial Wright test due to the prozone phenomenon, leading to a late diagnosis and delayed appropriate treatment.

## Case presentation

### Ethics statement

This case report’s steps were conducted according to the Helsinki Declaration and approved by the Ethics Committee of Fasa University of Medical Sciences (IR.FUMS.REC.1400.156). The patient filled out a written informed consent form.

**Fig 1:**
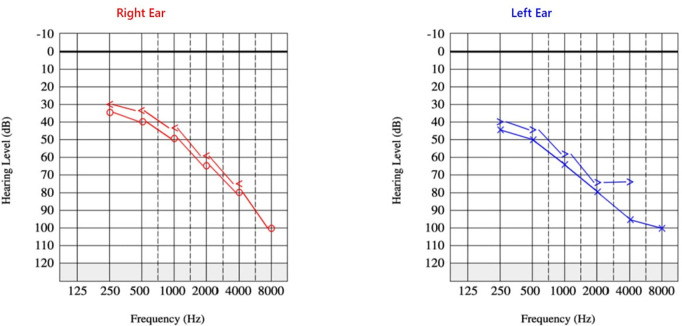
Audiogram after first admission. The plots show bilateral, downsloping sensorineural hearing loss (SNHL), which is more pronounced in higher frequencies.

We report the case of a 32-year-old Iranian rural man who presented with chief complaints of ataxia and fecal incontinence in November 2019 (Table 1). The symptoms started suddenly a week before admission, but gradually increased in frequency and severity. He had no vertigo, aphasia, nausea/vomiting, fever, chills, neck rigidity, or pain in any part of his body. His lower extremity sensation, tone, and power (right: 4/5, left: 3/5), as well as his deep tendon reflexes, were decreased. However, his upper extremities’ tone, power, sensation, and deep tendon reflexes were normal. Also, the finger-to-finger and finger-to-nose tests were completely abnormal, a clue to possible cerebellar ataxia. The visual field and acuity, as well as eye movements and pupillary reactions, were standard. Additionally, the patient reported no complaints of hearing loss or difficulty understanding others’ speech. The signs and symptoms did not improve during hospitalization and developed gradually within the four days of admission. The patient consented to be discharged to follow up on the diagnosis and treatment of his disease in outpatient visits. As he reported his condition after discharge, the paraplegia, urine-fecal incontinence, visual impairment, and Sensorineural hearing loss got worse; however, he did not go to any hospital or clinic due to the COVID-19 pandemic. Also, the patient suffered from a gradually increasing mood disorder after discharge. His appetite, sleep, and speech quantity decreased significantly in favor of depression disorder (Fig 1).

**Table 1:** Patient clinical summary.

Feature Category	Details
*Patient Profile*	**Age:** 32 years at initial presentation; **Sex:** Male; **Background:** Rural resident, occupational exposure to animals (dairy farm work)
**Initial Presentation (Nov 2019)**	
*Presenting Complaints*	Acute onset (1 week prior) of progressive ataxia and urinary/fecal incontinence. No fever, headache, nausea/vomiting, or neck rigidity was reported.
*Key Neurological Findings*	**Motor:** Lower extremity paraparesis (MRC Grade: R 4/5, L 3/5), decreased tone, and deep tendon reflexes (DTRs). Upper extremities normal; **Sensory:** Lower extremity sensation decreased; **Coordination:** Abnormal finger-to-finger and finger-to-nose tests (cerebellar ataxia); **Cranial Nerves:** Visual acuity/fields/movements/pupils normal. No hearing complaints.
*Initial Brucellosis Serology Serum*	Serum Wright Test: Negative Serum 2-Mercaptoethanol (2-ME) Test: Negative
*Initial Imaging (MRI)*	**Brain:** No pathological lesions; **Spinal** **Cord:** No signs of atrophy or active lesions. Mild thoracic cord narrowing was noted (interpreted as a chronic/stable finding). The vertebral column is regular except for minor osteophytes C3-C7.
**Interim Period (Nov 2019 - Sep 2021)**	
*Clinical Course*	Gradual worsening of paraparesis, incontinence, visual impairment, and onset of bilateral Sensorineural Hearing Loss (SNHL). Development of mood disorder (depression) with decreased appetite, sleep, and speech. Poor follow-up during the COVID-19 pandemic.
*Audiogram*	Bilateral down-sloping SNHL, especially in high frequencies (Fig 1A).
**Second Presentation (Sep 2021)**	
*Presenting Complaints*	Primary complaints of visual and hearing impairment (right eye vision rapidly deteriorated recently).
*Key Neurological Findings*	**Motor:** Worsened lower extremity weakness -severe paraparesis/paraplegia (MRC Grade: R 2/5, L 1/5). **Psychiatric:** Low appetite, mutism; Depression confirmed by psychiatrist. **Visual:** Impaired vision. **Auditory:** Bilateral SNHL confirmed by audiometry (Fig 1B).
*Brucellosis Serology (Serum)*	**Wright Test:** 1/2560 **2-ME Test**: 1/1280
*Cerebrospinal Fluid (CSF)*	**Analysis:** WBC 40/μL (30 Monocytes, 10 Neutrophils), RBC 10/μL, Glucose 41 mg/dL, Protein 32 mg/dL, LDH 48 U/L. **Brucellosis Tests:** Wright Test: Positive; 2-ME Test: Positive.
*Imaging (MRI)*	Brain and Spinal Cord: Stable compared to Nov 2019 MRI; no new or active lesions. Chronic mild thoracic narrowing unchanged.
*Electrophysiology*	EMG: Mild active denervation was noted in the upper and lower extremities.
**Treatment Initiated**	Intravenous Ceftriaxone (2g q12h) for 1 month, followed by oral Doxycycline (100mg q12h) and Rifampin (300mg q12h) for 5 months total therapy.
**Outcome (Last Follow-up: 2023)**	
*Neurological Status*	**Motor:** Significant improvement; walking with minimal ataxia. Lower extremity power R 5/5, L 4/5. Normal tone, sensation, DTRs. **Coordination:** Cerebellar tests normalized. Positive Romberg sign persisted (sensory ataxia).
*Visual Status*	Significant improvement; patient reported no difficulty seeing.
*Auditory Status*	There was a relative improvement in hearing, but SNHL did not fully resolve.
*Psychiatric Status*	Complete resolution of depressive symptoms, mood, and appetite.
*Imaging (MRI)*	Brain and Spinal Cord: Findings remained stable compared to previous scans (persistent mild chronic thoracic narrowing).

Since the patient reported a history of working on a dairy farm, a Wright test was immediately performed to rule out a possible diagnosis of brucellosis. But the test was entirely negative. A comprehensive laboratory test ruled out abnormal electrolytes, vitamins, glucose, thyroid, kidney, or liver function levels. The complete blood count showed a normal white blood cell (WBC) alongside low plasma levels of C-reactive protein (CRP) and erythrocyte sedimentation rate (ESR), ruling out the active phase of acute infection. Additionally, normal hemoglobin levels (13.5 g/dL), along with normochromic normocytic red blood cells, ruled out anemia. The random plasma glucose level was 101 mg/dL, ruling out hypoglycemia or diabetic ketoacidosis. Biochemical tests including K (4.8 mEq/L), Na (141 mEq/L), Cu (102 mg/dL), Ca (9.5 mg/dL), BUN (14 mg%), and creatinine (0.9 mg%) was all normal, ruling out the neurological complications of high or low level of electrolytes and Wilson’s Disease. The plasma level of vitamin B9 (10.6 ng/ml) and B12 (611 pg/ml) was normal, ruling out vitamin deficiency and Wernicke-Korsakoff syndrome. Also, liver function tests were normal, including aspartate aminotransferase (12 U/L) and alanine transaminase (13 U/L), total (0.5 mg/dL) and direct (0.1 mg/dL) bilirubin. The thyroid function test showed no hypo- or hyperthyroidism. The viral markers tests ruled out human T-lymphotropic virus type 1, human immunodeficiency virus, Cytomegalovirus, hepatitis A, B, and C virus infection. Further autoimmune tests, including anti-cardiolipin antibodies, anti-nuclear antibodies, and Perinuclear- anti-neutrophil cytoplasmic antibodies and Cytoplasmic- anti-neutrophil cytoplasmic antibodies, were performed and ruled out possible collagen vascular diseases.

**Fig 2:**
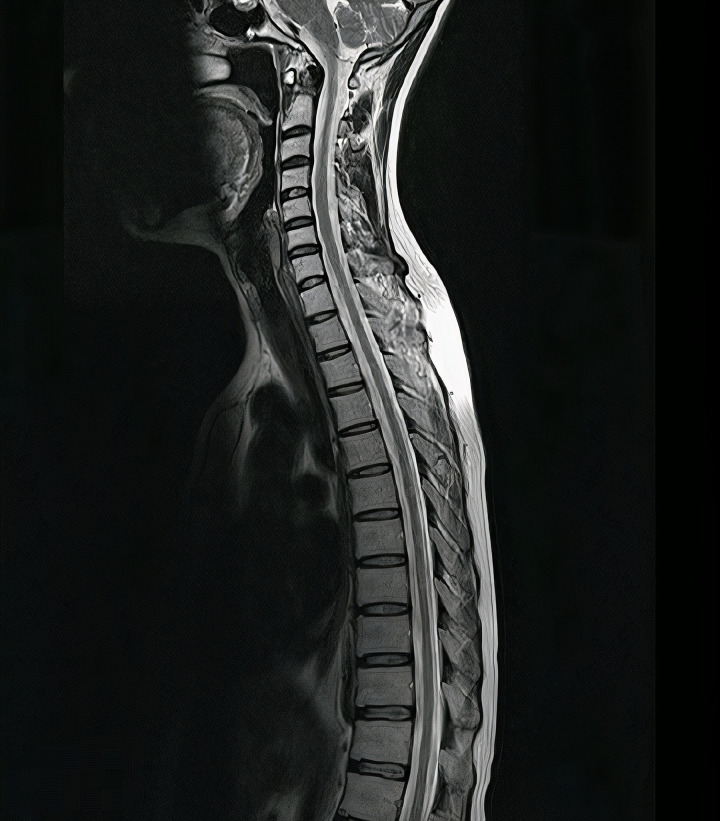
The sagittal view of T2-weighted Magnetic Resonance Imaging represented a mild decrease in spinal cord diameter without any other lesions at first admission.

After ruling out non-neurological pathologies, magnetic resonance imaging (MRI) was performed on the whole CNS, as shown in Fig 2 All vertebral bodies, spinous processes, and intervertebral discs of the column were completely intact, except small osteophytes observed in C3-C7, which had no compressive characteristics on the spinal cord. Also, the spinal cord had no signs of atrophy; however, it was mildly narrowed in the thoracic region, which was suggested as a chronic phenomenon with no sequelae. No pathological lesion was observed in the Brain MRI, including the brain stem, cerebrum, cortex, and base of the brain. For the second step, electromyography and nerve conduction studies were performed to rule out peripheral neuropathy and nerve root compression. Amyotrophic Lateral Sclerosis, and myositis. However, the results were normal. Therefore, the patient was referred to the neurologist’s office for further evaluation. Still, the patient consented to discharge himself, and due to the COVID-19 pandemic, he didn’t follow up on his medication anymore.

**Fig 3:**
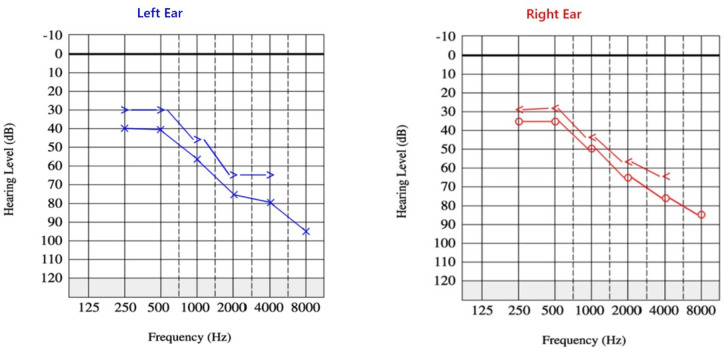
Audiogram after second admission. Findings demonstrate bilateral, downsloping SNHL identical to the results obtained after first admission, indicating stability of hearing loss.

In September 2021, he was referred to a hospital in Fasa with a chief complaint of visual and hearing impairment. The hearing loss gradually started over the past year and was diagnosed as bilateral SNHL (Fig 3).

**Fig 4:**
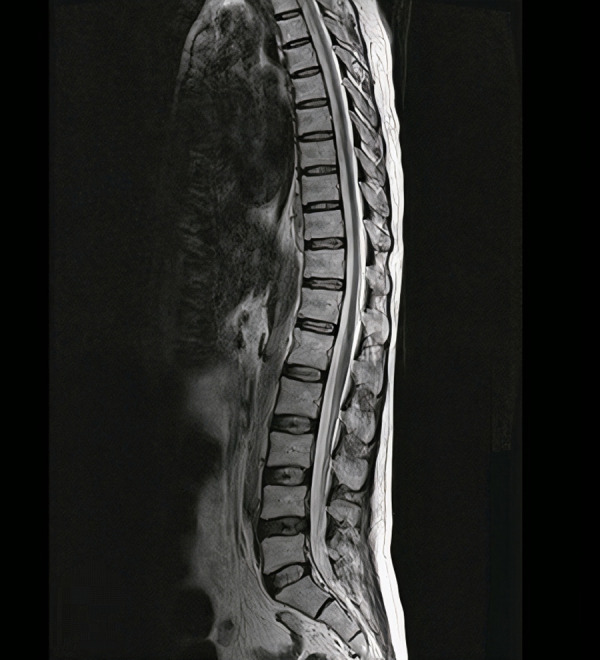
The sagittal view of T2-weighted Magnetic Resonance Imaging represented mild decreased spinal cord diameter without any other lesions at second admission.

Now, the visual impairment of the right eye has developed very fast in a few days. The paraplegia of the patient worsened (right: 2/5 left: 1/5). Also, he showed low appetite and mutism, which was confirmed as depression by a psychiatrist. Hence, the patient had a poor follow-up during the COVID-19 pandemic; all brucellosis tests were ordered at this admission again, including Wright (1/2560) and 2-ME (1/1280). The cerebrospinal fluid (CSF) sample also showed a positive Wright test. The CSF contained 40 WBC, 10 RBC, 30 monocytes, ten neutrophiles, 41 mg/dL glucose, 72 mg/dL protein, and 48 U/L lactate dehydrogenase. During this admission, the brain and spinal MRI was like the previous one without any new lesions or changes (Fig 4). Still, electromyography (EMG) showed mild active denervation in the upper and lower extremities this time, which explained the possible reason for the patient’s condition.

The patient was treated with ceftriaxone (2 gr, IV, Q12h) for one month, a doxycycline capsule (100 mg, Q12h), and a rifampin capsule (300 mg, Q12h) for five months. Following three weeks of antibiotic administration, significant improvement in urine-fecal incontinence, hearing loss, and visual impairment was diagnosed. Also, the patient’s mood and appetite disorder improved after a month.

**Fig 5:**
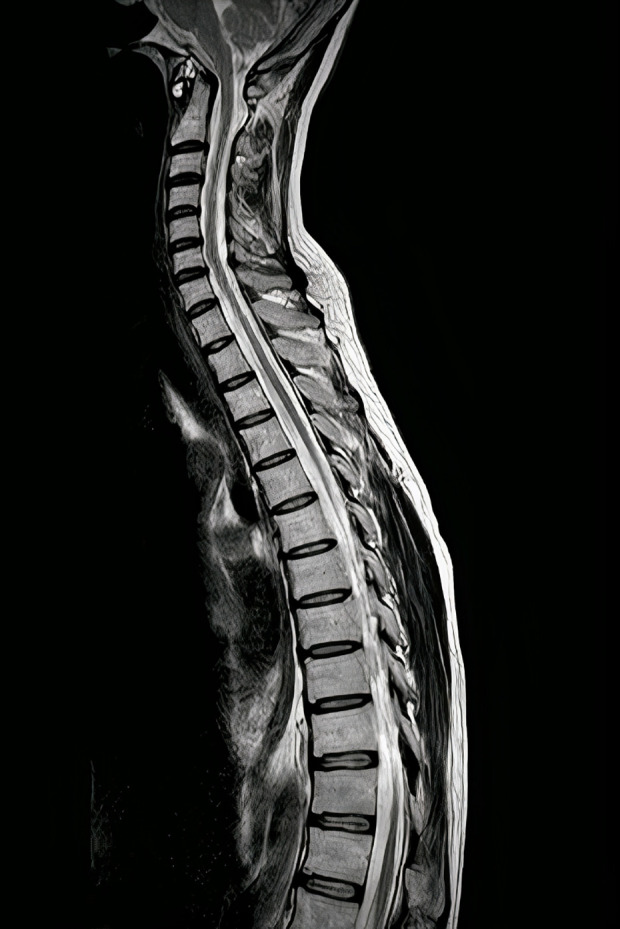
The sagittal view of T2-weighted Magnetic Resonance Imaging represented a mildly decreased spinal cord diameter without any other lesions at follow-up.

In his last follow-up visit in June 2024, the patient’s paraplegia completely disappeared, and his lower extremities’ power significantly improved (right 5/5 and left 4/5). His tone, sensation, and deep tendon reflex were completely normal. The finger-to-finger and finger-to-nose tests became utterly normal, but the patient could not maintain his balance with closed eyes, a positive Romberg sign. Also, his visual impairment has improved significantly, and he has no difficulty seeing near and far objects. The hearing loss became relatively better but did not completely disappear. Moreover, the psychiatric symptoms were repaired completely. The atrophy, diagnosed in his first admission, remained unchanged in his last Brain and Spinal MRI without any development or alleviation (Fig 5).

## Discussion

Brucellosis is an endemic disease in different areas of Iran. Although the incidence of brucellosis was reported to be significantly higher in the northern and northwest regions of Iran, the number of new cases of brucellosis has increased in the southern provinces of Iran [8]. Several factors, including the screening of contaminated animals, human and animal migration in Iran, socio-economic challenges, and poor hygiene, increase the probability of brucellosis infection [9]. In this study, we present a case of neurobrucellosis from Fars province, southwest Iran, a region known for its endemicity of brucellosis.

Commonly, brucellosis involves the hepatobiliary, skeletal, respiratory, and gastrointestinal systems [10]. However, life-threatening variations of brucellosis contaminate the CNS and result in serious complications such as brain abscess, meningitis, severe psychosis, encephalitis, and epidural abscess [11]. Most neurobrucellosis cases present with typical signs and symptoms such as fever, headache, nausea, and vomiting [5]. Our case showed no fever, headache, or nausea, decreasing the likelihood of CNS involvement. Also, the history of ataxia and urinary incontinence led the clinicians to rule out neurological diseases by imaging the spine and brain. Furthermore, the concurrent presentation of significant neurological deficits, such as paraplegia, ataxia, bilateral SNHL, visual impairment, and subsequent depression, represents a particularly complex and relatively uncommon constellation for neurobrucellosis. While each involvement, including hearing loss or psychiatric disturbances [12], was reported in previous observations, their simultaneous occurrence alongside severe motor deficits is less frequently reported, posing a significant diagnostic challenge. Also, previous studies reported that inflammation, vascular lesions, and neural involvements were joint in neurobrucellosis cases [6].

Interestingly, the first Wright test of our case was initially negative, with no typical history or symptoms of neurobrucellosis signs. Some previous studies addressed the prozone phenomenon as an essential reason for the false negative results in brucellosis diagnosis [13]. In these cases, very high antibody titers may lead to a misdiagnosis of brucellosis, thereby avoiding timely and appropriate management. Also, another study reported a seronegative neurobrucellosis who was presented atypical symptoms like depression and diplopia; however, the diagnosis was ultimately confirmed through CSF analysis or alternative serological tests [14]. These cases and ours strongly advocate for CSF examination in suspected neurobrucellosis within endemic areas, even when initial serum agglutination tests are negative, particularly if clinical suspicion remains high.

The process of neurological symptoms in our case started with paraplegia and ataxia, which was continued with gradually developing SNHL and visual impairment. The mechanism of complications development is still unclear. The direct damage of infection to the CNS or peripheral nerves would be the primary mechanism of these complications; however, our case presented no significant lesion in his Brain and spinal MRI. Previous studies have suggested a direct pro-inflammatory effect of neurobrucellosis on sensory and motor nerves [15]. Also, increasing intracranial pressure is another mechanism that would cause indirect damage to the nerves. Although visual impairment and SNHL are rare presentations in Neurobrucellosis cases, they can be observed in cases with late, inappropriate treatment or low compliance with antibiotics [16]. Since our case received his first antibiotic doses approximately two years after the first symptoms of neurobrucellosis, the complications developed very much and resulted in SNHL and impairment of vision. While SNHL and visual impairment are considered rarer complications, our patient’s significant bilateral hearing loss echoes findings in other case reports [17]. In our case, the marked improvement in vision and partial hearing recovery following delayed antibiotic therapy is noteworthy, suggesting that even late treatment initiation can yield benefits for cranial nerve involvements, although residual deficits may persist.

The presentation of mood disorder was another interesting complication of neurobrucellosis. Previously, other studies reported that some cases experience psychological disorders, such as behavior changes, agitation, impaired mood, and depression [18,19]. Moreover, psychiatric disorders would be the only presentation of neurobrucellosis. In another rare case report of a 17-year-old girl with seronegative neurobrucellosis, depression was the first presentation of infection while all tests were negative. In these situations, although negative Wright or Coombs Wright tests would mislead the clinical decision-making and detection of brucellosis, in patients who had sudden onset of mood disorders without reasonable mechanism, neurobrucellosis should be considered, especially in patients who live in endemic regions of brucellosis and have a suspicious contact with animals or their products [14]. Although depression was not the first presentation of neurobrucellosis in our case, it started after a while of disability progression. It became more severe when the patient had hearing loss and visual impairments. Similar to reports where psychiatric symptoms were prominent or even the initial manifestation [12], our patient developed significant depressive symptoms. However, in our case, the depression appeared secondary to the progression of debilitating neurological deficits rather than being the primary feature. The subsequent improvement in mood correlating with neurological recovery after treatment further supports a link, possibly reactive or directly related to CNS inflammation.

However, the appetite and mood of our patient improved after receiving antibiotics and alleviating his disabilities. This manner reveals the essential role of psychological concerns in neurobrucellosis patients, which should be approached in vulnerable cases.

The investigation of CNS imaging in our case revealed no significant lesions that could explain the condition. Commonly, meningeal enhancement, subcortical lesions, cortical nodules, massive or submissive edema in peripheral regions, and lesions in white or gray matter were mainly observed in neurobrucellosis cases [20,21]. Although MRI would be the most beneficial and efficient imaging modality for detecting typical lesions of neurobrucellosis, previous studies have reported that only more than 10% of cases presented with significant findings that provide a reasonable mechanism for clinical presentations [18]. Therefore, it is likely that no CNS lesions will be found while the patient is suffering from active neurobrucellosis. It is repeatedly recommended that neurobrucellosis be ruled out by collecting cerebrospinal fluid (CSF) specimens, especially in highly suspicious cases. In our case, although brucellosis infected the CNS, no signs of damage to the vertebral column, spinal cord, or brain were found. The Wright plasma test was unreliable due to the prozone phenomenon. Consequently, patients would benefit significantly if the Coombs Wright test, a more sensitive and specific test, were performed when the routine plasma test was negative [22]. Additionally, in patients with neurological symptoms of unclear etiology, a lumbar puncture would help obtain a more specific specimen for the Wright test in endemic regions for brucellosis [22]. Interpreting serological results for neurobrucellosis, including standard agglutination tests, like the Wright & 2ME tests, warrants careful consideration. While these tests are commonly used, the sensitivity and specificity of serum standard agglutination tests would fluctuate depending on factors such as the stage of the infection and the patient’s immune status [23,24]. Specificity might be compromised by cross-reactivity with other microorganisms. Additionally, test sensitivity can decline in chronic disease stages or, as illustrated by our patient, exceptionally high antibody concentrations.

The involvement of the peripheral neural system is another possible mechanism of the presentation of disabilities in our case. Interestingly, although the patient had significant ataxia (in favor of cerebral), paraplegia, and urine fecal incontinence, there were no abnormalities in his EMG findings at first admission. Also, the patient had no severe chronic complications of neurological disabilities, such as typical ulcers in the lower extremities, muscular atrophy, deformity of joints, or atypical pressure ulcers induced by spasms related to upper motor neuron damage [25,26].

However, two years after the infection developed and he had impaired visual and SNHL, the second EMG reported mild denervation in his upper and lower extremities, which did not have enough rationale to explain the clinical findings. Polyneuropathies have several differential diagnoses that were evaluated in our case. The routine daily plasma glucose (fasting and postprandial) was monitored, and diabetic polyneuropathy was ruled out. However, the patient was young and had no history of tingling or elevated plasma glucose levels before admission [27]. Various viral markers associated with polyneuropathy were checked, but all markers were negative in all admissions [28]. Other possible pathologies are autoimmune diseases, which were ruled out by checking known autoantibodies that cause polyneuropathy [29,30]. Eventually, biochemical tests revealed no significant dysfunction of the liver, kidney, or thyroid, which would result in polyneuropathies [31–33].

Investigating the central nervous system (CNS) and peripheral nervous system, alongside a comprehensive approach to possible differential diagnoses, revealed no disease that would be accompanied by neurobrucellosis in our patient. On the other hand, we found no clinical or paraclinical findings that explain the existing neurological disorders, except for neurobrucellosis, without any visible damage to the CNS or remarkable lesions in peripheral nerves, which account for these manifestations.

Timely administration of antibiotics, including Doxycycline, rifampicin, and third-generation cephalosporins with appropriate dosage, significantly alleviates symptoms [34]. Previous cases achieved the best outcomes six weeks after antibiotic therapy [35]. Although our case was deprived of early treatment, the neurological recovery was acceptable after a long period of symptoms. While the patient achieved more power in his lower extremities and could walk with minimal ataxia, he still had trouble with hearing loss. Therefore, even late antibiotic therapy has significant benefits for cases of Neurobrucellosis. On the other hand, several factors contributed to the patient experiencing minimal symptoms after antibiotic therapy. First, appropriate doses with standard duration were utilized to treat the infection. Also, the patients had no significant lesions in the CNS, which would cause no further irreversible damage [34,35].

## Conclusion

We report a rare case of neurobrucellosis presented with depression, visual impairment, bilateral SNHL, paraplegia, and an initial false negative Wright test due to the prozone phenomenon. Hence, the prozone phenomenon in the Wright test should be considered in regions where brucellosis is endemic.
